# Top 10 dietary strategies for atherosclerotic cardiovascular risk reduction

**DOI:** 10.1016/j.ajpc.2020.100106

**Published:** 2020-11-19

**Authors:** Geeta Sikand, Tracy Severson

**Affiliations:** a University of California, Irvine, USA; b Oregon Health and Science University, USA

**Keywords:** DASH, Mediterranean, Plant based/vegetarian/vegan dietary patterns, Cardiovascular risk reduction, Lipoproteins, Low-carbohydrate diet, Medical nutrition therapy, Obesity, Triglycerides, Weight loss, Very-low-carbohydrate diet

## Abstract

Poor dietary quality has surpassed all other mortality risk factors, accounting for 11 million deaths and half of CVD deaths globally. Implementation of current nutrition recommendations from the American Heart Association (AHA), American College of Cardiology (ACC) and the National Lipid Association (NLA) can markedly benefit the primary and secondary prevention of atherosclerotic cardiovascular disease (ASCVD). These include: 1) incorporate nutrition screening into medical visits; 2) refer patients to a registered dietitian nutritionist (RDN) for medical nutrition therapy, when appropriate, for prevention of ASCVD; 3) follow ACC/AHA Nutrition and Diet Recommendations for ASCVD prevention and management of overweight/obesity, type 2 diabetes and hypertension; 4) include NLA nutrition goals for optimizing low-density lipoprotein cholesterol (LDL-C) and non-high-density lipoprotein cholesterol (non-HDL-C) and reducing ASCVD risk; 5) utilize evidence-based heart-healthy eating patterns for improving cardiometabolic risk factors, dyslipidemia and ASCVD risk; 6) implement ACC/AHA/NLA nutrition and lifestyle recommendations for optimizing triglyceride levels; 7) understand the impact of saturated fats, trans fats, omega-3 and omega-6 polyunsaturated fats and monounsaturated fats on ASCVD risk; 8) limit excessive intake of dietary cholesterol for those with dyslipidemia, diabetes and at risk for heart failure; 9) include dietary adjuncts such as viscous fiber, plant sterols/stanols and probiotics; and 10) implement AHA/ACC and NLA physical activity recommendations for the optimization of lipids and prevention of ASCVD. Evidence on controversies pertaining to saturated fat, processed meat, red meat, intermittent fasting, low-carbohydrate/very-low-carbohydrate diets and caffeine are discussed.

## 1 Introduction

Cardiovascular disease (CVD) remains the leading cause of morbidity and mortality in the United States and globally [[1], [2], [3], [4], [5]]. Poor dietary quality has surpassed all other mortality risk factors, accounting for 11 million deaths and ~50% of CVD deaths globally [5]. Recently, deaths from CVD have increased in the middle age (45–64 years) cohort in the United States. This has been attributed to sub-optimal diets leading to increases in overweight/obesity, dyslipidemia, hypertension, pre-diabetes, diabetes, and metabolic syndrome [5]. Atherosclerotic cardiovascular disease (ASCVD) also remains the leading cause of death among several racial/ethnic groups in the United States, with an estimated cost of >$200 billion annually in healthcare services, medications, and lost productivity [[1], [2], [3], [4], [5]]. This increased cost is mostly attributable to poor diet quality due to suboptimal implementation of prevention strategies and uncontrolled ASCVD risk factors in many adults [[1], [2], [3], [4], [5]]. The cornerstone of preventing ASCVD, heart failure, and atrial fibrillation is to promote a healthy lifestyle throughout life [[1], [2], [3], [4], [5]].

To attenuate the high global burden of disease, the American Heart Association (AHA) 2030 goals [6] focus has shifted from managing heart disease to the prevention of heart disease. The AHA now aims to ensure that prevention-related interventions for heart disease and stroke events effectively reach the vulnerable populations, communities and the healthcare and public health systems [6]. The new AHA emphasis is to support healthful behavior changes, specifically diet and physical activity for prevention and treatment of obesity, diabetes mellitus, hypertension, hyperlipidemia and CVD [6].

Implementation of dietary strategies that promote healthier dietary patterns to reduce ASCVD risk is critical. To help facilitate these necessary lifestyle changes, the top ten dietary strategies to reduce ASCVD risk are summarized below.

## 2 Strategy 1: Incorporate nutrition screening into medical visits to assess dietary quality and determine need for referral to a registered dietitian nutritionist (RDN)

The AHA recommends nutrition screening at the point of care, stating that it is critical to assess patients’ diet quality and discuss with members of the healthcare team to reduce the incidence and improve the management of CVD [7]. It is noteworthy that nutrition screening or counseling is not a standard of practice during routine medical visits, due to numerous barriers including lack of training and knowledge, lack of time, lack of reimbursement, and absence of validated rapid diet screener tools coupled with clinical support to identify actionable modifications [7]**.** An AHA scientific statement addressed the use of dietary screening tools. Of the 15 tools reviewed, the three that met the most theoretical and practice-based validity criteria were the Mediterranean Diet Adherence Screener (MEDAS) and its variations; the modified, shortened Rapid Eating Assessment for Participants (REAP), and the modified version of the Starting the Conversation Tool, though the authors noted that the Powell and Greenberg Screening Tool was the least time consuming [7]**.** A recent study examined the validity and reliability of an instrument developed to reflect current evidence-based dietary recommendations advocated to reduce CV risk in Cardiac Rehabilitation (CR) patients (N ​= ​108) [**8**]. Two dietary assessments were administered: Picture Your Plate (PYP) and a reference instrument, the Harvard/Willett Food Frequency Questionnaire (HWFFQ). Crude and adjusted Spearmen correlation coefficients between the PYP and 3 indexes of dietary quality were AHEI-2010 (0.71–0.72), DASH (0.70–0.71), and aMED (0.52–0.58) (*P* ​< ​0.0001, all comparisons). Agreement of tertiles comparing PYP and AHEI-2010 was 67% and the score in opposite tertiles was 6%. The weighted kappa value (κw) ​= ​0.71. The test-retest ICC was 0.91 (95% CI, 0.85–0.93; n ​= ​91). The authors concluded that the PYP is a valid and reliable dietary assessment tool for use in CR settings [8].

Nutrition screening should begin a conversation about the impact of diet on cardiovascular health. Patients whose screening results indicate poor dietary quality, in particular those with dyslipidemia (high low-density lipoprotein cholesterol [LDL-C], high non-high-density lipoprotein cholesterol [non-HDL-C] and high triglycerides [TG]) and/or other cardiometabolic risk factors (diabetes, pre-diabetes, hypertension, overweight/obesity and metabolic syndrome), should be referred to the registered dietitian nutritionist (RDN) for multiple visits for medical nutrition therapy [[2], [3], [4],[7], [14]].

Evidence is strong that patients referred to the RDN for medical nutrition therapy for the treatment of dyslipidemia and cardiometabolic risk factors achieve significant improvements [[2], [3], [4],[9], [10], [11], [12], [13], [14]]. Sikand and colleagues conducted a systematic review and meta-analysis of 5704 patients (34 primary studies) with dyslipidemia and cardiometabolic risk factors and reported that multiple visits with a RDN led to significant improvements in LDL-C, TG, body mass index (BMI), BP and glycosylated hemoglobin (HbA1c) and subsequent cost savings from a reduction in medications for dyslipidemia, BP and diabetes. Similar outcomes have been reported in several dietitian intervention outcome studies [[2], [3], [4],^9^,^10^,[12], [13], [14]].

## 3 Strategy 2: Refer patients to an RDN for medical nutrition therapy, when appropriate, for prevention of ASCVD

In addition to an indication of poor dietary quality based on the results of a validated screening tool, the following criteria should generate a referral to an RDN for medical nutrition therapy to improve cardiometabolic risk factors for prevention of ASCVD [4,9,10,[12], [13], [14],[24], [25], [26], [27]]:

• Excess weight, defined as:
  Overweight body mass index (BMI) 24.9–29.9 ​kg/m^2^
  BMI Class 1 obesity 30–34.9 ​kg/m^2^, Class II obesity 35–39.9 ​kg/m^2^ and Class III obesity >40 ​kg/m^2^
  Abdominal obesity: waist circumference >35 inches in men, >32 inches in women
• Abnormal LDL-C, TG and/or non-HDL-C
• Uncontrolled hypertension
• Poorly-controlled diabetes, defined by HbA1c ​> ​7 ​mg/dL
• Pre-diabetes, defined by abnormal fasting glucose ≥100 ​mg/dL

Medical nutrition therapy, or personalized nutrition counseling, is a cornerstone of practice for RDNs as they target diet and lifestyle behavior change while providing support and accountability for long-term success [[2], [3], [4],[9], [10], [11], [12], [13], [14]]. While RDNs use the standardized systematic framework of the nutrition care process (NCP) to guide nutrition care [9], nutrition care decisions are made on an individual basis according to specific individual needs, values and clinical experience in combination with the best available evidence. An RDN personalizes each patient’s eating plan by tailoring their usual dietary intake, integrating a heart-healthy dietary pattern while considering lifestyle, food preferences, culture/religion/ethnicity, and economic and psycho-social needs [[2], [3], [4],[9], [10], [11], [12], [13], [14]]. Medical nutrition therapy by the RDN includes four components: 1) nutrition assessment, 2) nutrition diagnosis, 3) nutrition intervention and monitoring and 4) evaluation and communication with the referring health care provider [[10], [11], [12], [13], [14], [9]].

## 4 Strategy 3: Follow ACC/AHA Nutrition and Diet Recommendations for ASCVD Prevention and Management of Overweight/Obesity, Type 2 Diabetes (T2DM) and Hypertension.

• **ACC/AHA Nutrition and Diet Recommendations for Prevention of ASCVD** [2]**:**
  • A diet emphasizing intake of vegetables, fruits, legumes, nuts, whole grains, and fish is recommended to decrease ASCVD risk factors.
  • Replacement of saturated fat with dietary monounsaturated fatty acids (MUFA) and polyunsaturated fatty acids (PUFA) reduces ASCVD risk.
  • A diet lower in sodium and cholesterol decreases ASCVD risk.
  • As a part of a healthy diet, it is reasonable to minimize the intake of processed meats, refined carbohydrates, and sweetened beverages to reduce ASCVD risk.
  • As a part of a healthy diet, the intake of trans fats should be avoided to reduce ASCVD risk.
➢ **ACC/AHA Nutrition and Diet Recommendations for Adults with Overweight and Obesity** [2]**:**
  • In individuals with overweight and obesity, weight loss is recommended to improve the ASCVD risk factor profile.
  • Counseling and comprehensive lifestyle interventions, including calorie restriction, are recommended for achieving and maintaining weight loss in adults with overweight and obesity.
  • Calculating BMI is recommended annually or more frequently to identify adults with overweight and obesity for weight loss considerations.
  • It is reasonable to measure waist circumference to identify those at higher cardiometabolic risk.

Adults diagnosed with obesity (BMI ≥30 ​kg/m2) or overweight (BMI ​= ​25–29.9 ​kg/m^2^) are at an increased risk of ASCVD, heart failure, and atrial fibrillation versus normal weight individuals [[1], [2], [3], [4]]. Waist circumference measurement is recommended in all patients with a BMI <35 ​kg/m2. Weight loss of 5%–10% of initial weight, achieved through comprehensive lifestyle intervention, has been shown to improve blood pressure (BP), delay the onset of T2DM, improve glycemic control in T2DM, and improve the lipid profile [[1], [2], [3], [4]], while multidisciplinary lifestyle intervention programs, including counseling by an RDN, FDA–approved drugs, and bariatric surgery, are associated with significant reductions in waist circumference, lipids, A1c and BP [2], [4], [9], [10], [11], [12], [13], [14], [15], [16]. Ethnic differences are reported in in BMI and waist circumference thresholds associated with cardiometabolic risk [2], [19], [20], [21], [22], [23], [24].

➢ **ACC/AHA Nutrition Recommendations for Adults with T2DM** [2]**:**
  • For all adults with T2DM, a tailored nutrition plan focusing on a heart-healthy dietary pattern is recommended to improve glycemic control, achieve weight loss, if needed, and improve other ASCVD risk factors [2]. A heart-healthy dietary pattern is a key intervention in the treatment of T2DM [24,25]. Weight loss can be essential for treatment of T2DM, and dietary recommendations should be adjusted to achieve meaningful weight loss, if needed [[1], [2], [24], [25], [3], [4],^24^,^25^]. The Mediterranean, DASH, and plant-based (vegetarian/vegan) diets have all been shown to achieve weight loss and improve glycemic control in T2DM [[1], [2], [3], [4],^24^,^25^].
• **ACC/AHA Nutrition Recommendations for Prevention and Treatment of Hypertension** [2], [41]**:**
  • **Weight loss:** The best goal is ideal body weight, but aim for at least a 1-kg reduction in body weight for most adults who are overweight. Expect about 1 ​mm Hg reduction in systolic blood pressure (SBP) for every 1-kg reduction in body weight. Expected impact on SBP: −5 ​mm Hg in hypertensives and −2/3 ​mm Hg in normotensives [41].
  • **DASH (Dietary Approaches to Stop Hypertension) diet:** Consume a diet rich in fruits, vegetables, whole grains, and low-fat dairy products, with reduced content of saturated and total fat. Expected impact on SBP: −11 ​mm Hg in hypertensives and 3 ​mm Hg in normotensives [41].
  • **Reduced intake of sodium:** Optimal goal is ​< ​1500 ​mg/d, but aim for at least a 1000-mg/d reduction in most adults. Expected impact on SBP ​= ​−5/6 ​mm Hg in hypertensive and −2/3 ​mm Hg in normotensive individuals [41].
  • **Increased intake of potassium:** Aim for 3500–5000 ​mg/d, preferably by consumption of a diet rich in potassium. Expected impact on SBP ​= ​−4/5 ​mm Hg in hypertensive and −3 ​mm Hg in normotensive individuals [41].
  • **Alcohol consumption:** In individuals who drink alcohol, reduce alcohol to [41]:
    Men: ≤2 drinks daily
    Women: ≤1 drink daily

Expected impact on SBP ​= ​−3 ​mm Hg in hypertensive and −2 ​mm Hg in normotensive individuals. In the United States, one “standard” drink contains roughly 14 ​g of pure alcohol, which is typically found in 12 oz of regular beer (usually about 5% alcohol), 5 oz of wine (usually about 12% alcohol), and 1.5 oz of distilled spirits (usually about 40% alcohol). Drinking in excess can lead to alcoholism, high blood pressure, obesity, stroke, breast cancer, suicide and accidents [[1], [2], [3], [4]]**.**

## 5 Strategy 4: Include NLA nutrition goals for optimizing LDL-C and non-HDL-C and reducing ASCVD risk [4].

The NLA nutrition goals for optimizing LDL-C, non-HDL-C and reducing ASCVD risk are as follows [4]**:**

• Achieve weight loss of 5–10% of body weight if overweight.
• Reduce saturated fat intake to <7% of total energy and dietary cholesterol to <200 ​mg/day.
• Avoid trans fats.
• Reduce intake of added sugars to <10% of total energy.
• Follow a heart-healthy dietary pattern with a focus on plant-based protein.
• Increase intake of viscous fiber to 5–10 ​g/day and plant sterols/stanols to 2 ​g/day.

## 6 Strategy 5: Utilize evidence-based heart-healthy eating patterns for improving cardiometabolic risk factors, dyslipidemia and ASCVD risk

The adoption of a heart-healthy eating pattern can have far-reaching cardiovascular benefits [1], [2], [3], [4], [26], [27], [28], [29], [30], [31], [32], [33]. Scientific evidence from randomized controlled trials (RCT) revealed that each 1% reduction in LDL-C or non-HDL-C is associated with a 1% decrease in coronary heart disease (CHD) event risk over 5 years [[1], [2], [3], [4]]. Weight loss of 5–8 ​kg, if sustained, results in mean LDL-C reduction of 5 ​mg/dL and an increase in HDL-C of 2–3 ​mg/dL, while a 3 ​kg weight loss reduces TG by 15 ​mg/dL [[1], [2], [3], [4]]. In addition to direct benefits on lipids and weight, heart-healthy eating patterns are also associated with improved non-traditional risk factors including markers of inflammation, insulin resistance, oxidative stress and thrombogenicity [[1], [2], [3], [4],[28], [29], [30], [31]].

### 6.1 Components of any US-style heart-healthy eating pattern [[1], [2], [3], [4],[28], [29], [30], [31]]:

• Inclusion of healthful foods and beverages within an appropriate calorie level to achieve and maintain an optimal body weight. Healthful foods include:
  A variety of vegetables from all of the subgroups—dark green, red and orange, legumes (beans and peas), starchy, and other
  Fruits, especially whole fruits
  Grains, at least half of which are whole grains
  Fat-free or low-fat dairy, including milk, yogurt, cheese, and/or fortified soy beverages
  A variety of protein foods, including seafood, lean meats, poultry, eggs, legumes (beans and peas), nuts, seeds, and soy products
  Non-tropical oils
• Limited intake of saturated fat, refined grains, red and processed meats, sodium, and sugar-sweetened foods and beverages.
• Avoidance of trans fat

### 6.2 Evidence-based healthy US-style food patterns:

• **DASH dietary pattern:** The original DASH dietary pattern as well as the higher unsaturated fat-DASH pattern improve blood pressure, blood lipids, and ASCVD risk. The DASH dietary patterns (Table 1) are high in vegetables, fruits, whole grains, low or non-fat dairy, seafood, skinless poultry, legumes and nuts; moderate in alcohol (for adults); low in red and processed meats, refined grains and sugar-sweetened foods and beverages [[1], [2], [3], [4],[28], [29], [30], [31]].

**Table 1:** American Heart Association Recommended Dietary Pattern based on Dietary Approaches to Stop Hypertension feeding trials (DASH) [30∗].

Food Group	Amount/Day	Amount/Week
Fruits: fresh/frozen/canned (unsweetened preferred (cups)	2	14
Vegetables: fresh/frozen/canned (cups)	2½	10½
Dark green vegetables (cups)∗∗		1½
Red/orange vegetables (cups)∗∗		5½
Beans and peas (cups)∗∗		1½
Starchy vegetables (cups)∗∗		5
Other vegetables (cups)∗∗		4
Grains; emphasize whole grains/high in dietary fiber (oz eq/day)	6	42
Whole grains (oz eq/day)	3	21
Other grains (oz eq/day)	3	21
Protein foods (oz eq)	5½	39
Lean meat, poultry, eggs, oz eq		26
Fish, preferably oily fish, oz eq		8
Nuts, seeds, legumes, oz eq∗	1	5–7
Dairy: fat free or low fat, cups	3	21
Oils: unsaturated sources (g/day [Tbsp])	45 [3]	415 [21]
Fiber (g)	31	217
Solid fats (g [% of total kcal])	13 [6]	91(6)
Added sugars (g [kcal])	25 (100)	175 (700)
Sodium (mg)∗∗∗	<2300 ​mg	<16,100

∗Based on 2000 ​kcal; adjustments should be made to meet energy needs.

∗∗Indicates no daily requirement, rather weekly intake as noted.

∗∗∗ Overall goal for sodium is 1500 ​mg/day, but gradual reduction to achieve 2300 ​mg/day may be more realistic; average US intake for adults is 3500 ​mg/day.

Note: The American Heart Association’s basic dietary pattern is similar to the Dietary Approaches to Stop Hypertension (DASH) and MyPlate, with the following caveats.

•DASH restricts sweets to five per week rather than an added sugar limit in teaspoons.

•DASH allows a lower range of total fat, with a slight increase in meat (rather than 45 ​g of oil, DASH allows 30 ​g–45 ​g).

•On a 2000-kcal diet, DASH includes 6 oz of meat/fish/poultry. Vegetable protein sources are encouraged.
• **Mediterranean-style dietary pattern:** Like DASH, the Mediterranean dietary pattern (MedDiet) is high in fruits, vegetables, whole grains, legumes, unsalted nuts and seeds and olive oil; low to moderate in fish, skinless poultry, low-fat dairy products and red wine (in individuals consuming alcohol); and low in red meat [[1], [2], [3], [4],[28], [29], [30], [31], [32]]. The MedDiet eating pattern is also high in MUFA, PUFA, polyphenols, flavonoids, phytosterols and fiber, all of which contribute to reduced risk of CVD and DM [[1], [2], [3], [4],[28], [29], [30], [31], [32]]. In the republished PREDIMED study [32]**,** 5859 adults, ages 55–80 years with T2DM or at least 3 major risk factors (no CVD), showed significantly lower rates of major CV events with an energy-unrestricted MedDiet PLUS extra-virgin olive oil OR mixed unsalted mixed nuts versus a reduced-fat diet (control group) [34]. Results were similar even after the omission of 1588 participants whose study-group assignments were known or suspected to have departed from the protocol [32].
• **Vegetarian, vegan and flexitarian plant-based dietary patterns**: Vegetarian and vegan diets are typically high in fruits, vegetables, grains, legumes, and nuts, while limiting or avoiding animal products (lacto-ovo-vegetarians consume dairy products and eggs, while vegans consume only plant foods) [1,28]. Vegetarians typically have a higher intake of fiber, carbohydrate, potassium, magnesium, folate, n-6 fatty acids, non-heme iron and vitamin C than non-vegetarians [28]. Studies have shown that vegetarians and vegans, compared to omnivores, have lower BMI, LDL-C, glucose, hsCRP and TMAO levels, along with a lower incidence of mortality (CVD and overall) [[35], [36], [37], [38], [39], [40]].

Studies have also demonstrated that “flexitarian” diets – primarily plant-based diets with limited intake of animal products – also confer cardiovascular benefits. A large prospective cohort [36] of 416,104 men and women (median (SD) ages were 62.2 (5.4) years for men and 62.0 (5.4) years for women) reported that replacement of 3% energy from animal protein with plant protein was inversely associated with overall mortality (risk decreased 10% in both men and women) and cardiovascular disease mortality (11% lower risk in men and 12% lower risk in women) [36]. Similarly, the development of a healthful plant-based diet index (healthful-PDI), in which healthy plant foods receive positive scores and less healthy plant foods and animal foods receive reverse scores, has been used to demonstrate that greater adherence to healthy plant-based diets is associated with a decreased incidence of CVD [37].

• **Other evidence-based cardioprotective dietary pattern**s: The 2015–2020 Dietary Guidelines for Americans (DGA) recommendation for optimal carbohydrate intake is 45%–65% of energy intake [28]. However, some ultra-low-fat/very-high-carbohydrate diets like the Esselstyn [42] and the Ornish diets [43] reduce CVD risk and reverse heart disease [42,43]. Furthermore, the habitual very-high-carbohydrate/very-low-fat-diets consumed by Okinawan Japanese [44] and the Tsimani Indians [45] in South America have shown a reduced rate of ASCVD and an increase in longevity. These diets, although high in total carbohydrate, are low in refined carbohydrates [[42], [43], [44], [45]].

### 6.3 Evidence on additional popular eating plans:

• **Low-carbohydrate and very-low-carbohydrate (including ketogenic) diets:** Restricting carbohydrates (CHO) as a means of improving ASCVD risk factors could be beneficial for some in the short-term, while a high-quality moderate-CHO/low-fat diet defined as 26–44% of total daily energy (TDE) from CHO (98–168 ​g/d) and 25–35% TDE from fat may work better for others. The current popular ketogenic diet is very low in CHO (20–50 ​g/d or 5–10% TDE) and high in fat (70–80% TDE), and may lower TG, blood glucose and body weight. People with overweight/obesity who also have diabetes and/or elevated TG may benefit from following a hypocaloric low-CHO and very-low-CHO diet for two to six months, as they have been shown to result in greater short-term weight loss than hypocaloric high–CHO–low-fat diets (CHO 45–65% TDE and fat 25–35%). However, for the long-term (>6 months), the results from the low-CHO diet (10–25% TDE) were not sustained and were similar to the results of the high-CHO/low-fat diet (CHO 45–65% TDE and fat 25–35%) [17], [47], [48].

Very-low-CHO diets are difficult to maintain and are not nutritionally adequate, eliminating fiber-rich starchy vegetables, most fruits, legumes and whole grains, all foods that are associated with a reduced cardiometabolic risk [17]. Long-term dietary patterns with low-CHO intake along with high animal-derived fat/protein intake may increase LDL-C and are associated with increased cardiac and non-cardiac mortality [17], [47], [48]. Further, the vegetarian/vegan, DASH and Mediterranean dietary patterns also lead to improvements in BMI, TG, HDL-C, and HbA1c levels in individuals with T2DM compared to low-CHO diets [17].

The Atherosclerosis Risk in Communities study (ARIC) (n ​= ​15,428) [49] as well as a meta-analysis with data from ARIC plus 7 multi-national prospective studies (n ​= ​432,179) examined the association between CHO intake and all-cause mortality. The ARIC analyses demonstrated that both low (<40% daily energy intake) and high CHO (>70% daily energy intake) diets were associated with a higher risk of mortality (20% and 23%, respectively) and 50–55% TDE was associated with the lowest risk of mortality [49]. When animal-based protein or fat was substituted for CHO, the associated risk of mortality increased by 18% whereas mortality decreased by 18% when CHO was replaced by plant-based protein or fat [49].

Low-CHO dietary patterns favoring animal-derived protein and fat intake from sources such as lamb, beef, pork, and chicken, have been associated with higher mortality, whereas those that favored plant-derived protein and fat intake, from sources such as vegetables, nuts, peanut butter, and whole-grain breads, were associated with lower mortality, suggesting that the source of food notably modifies the association between CHO intake and mortality [49]. Association between CHO restriction and increased mortality could be attributed to a reduced intake of vegetables, fruits, and grains, and an increased intake of animal-based protein, which results in reduced levels of dietary bioactive nutrients (e.g., free fatty acids, protein, fiber, minerals, vitamins, and phytochemicals). Higher CHO intake could also be associated with lower socio-economic status and consumption of lower-quality (refined grains) carbohydrate foods [48,49].

These low-carbohydrate and very-low-carbohydrate dietary patterns are not consistent with nutrition recommendations by the ACC/AHA dietary guidelines and other professional organizations. The NLA Scientific Statement [17] recommends that, after a careful patient-provider discussion, if the obese patient chooses to follow a severely restrictive carbohydrate diet for weight loss, it should be limited to short periods (2–6 months) followed by a transition to a healthy dietary pattern for the long-term with adequate intake of fiber-rich carbohydrate foods and inclusion of plant-based proteins and unsaturated fats for nutritional adequacy and to prevent ASCVD. Further, medical monitoring should also include routine lipid testing and patients should participate in a comprehensive multidisciplinary lifestyle management program [17].

• **Intermittent Fasting:** Intermittent fasting (IF) is an umbrella term for various fasting regimens, including time-restricted feeding (TRF), alternate-day fasting, and the 5:2 (five days on IF and two days off IF) diet, all of which incorporate short-term fasts as a means of losing weight or improving health [50,51]. Most IF research involves animal studies, and, while they have shown promising results in the prevention and management of chronic metabolic diseases, human studies are very limited in number and duration, with very few studies lasting over 26 weeks [52].

In studies comparing IF to continuous energy restriction (CER) in adults with overweight or obesity with weight loss as the primary outcome, subjects have lost an average of 3–8% body weight, with no significant differences reported between the IF and CER groups in most studies [53]. The impact of IF on cardiometabolic health, including total and LDL-C, TG, and fasting glucose, has been modest and similar to the changes reported in subjects using CER, indicating that the weight loss is the likely driving force behind the improvements in CV risk markers [53].

Studies suggest that IF models are safe for most healthy adults, and may help with weight loss about as much as other calorie-restricted eating plans. For people looking to improve their CV health, an early TRF regimen (with the eating period earlier in the day) may provide some benefit over other IF regimens such as alternate-day fasts or late TRF (with the eating period later in the day/evening), though there is not yet enough evidence to recommend these plans [54].

## 7 Strategy 6: Implement ACC/AHA/NLA nutrition and lifestyle recommendations for optimizing TG levels

Hypertriglyceridemia (TG 200–499 ​mg/dL) is common in the United States, whereas more severe TG elevations (very high TG, ≥500 ​mg/dL) is less common. However, both are becoming increasingly prevalent in the United States and attributed to the growing rates of obesity and T2DM [[1], [2], [3], [4]]. Classification of TG by normal, borderline high, high and very high levels is noted in Table 2.

• Achieve weight loss of approximately 5–10% of body weight, which can lower TG 20% as well as reduce the risk of developing metabolic syndrome (MetS) and T2DM [[2], [3], [4]]. One study examined 125 patients with MetS over 17 weeks and reported a 45% reduction in TG with a 15% weight loss on a very low-calorie diet [55]. The magnitude of reduction in TG is related to the magnitude of weight loss, but even a small (3–5%) reduction in body weight can lower TG [[1], [2], [3], [4]]. A strong association has been demonstrated between TG and weight, elevated BMI, and visceral fat [[2], [3], [4]].
• Avoid excessive intake of carbohydrate, especially refined carbohydrates, e.g., sugar and sweets. When 60–65% of energy is consumed as carbohydrate, it leads to upregulation of very low-density lipoprotein (VLDL) secretion [[2], [3], [4]].
• Choose vegetable oils over solid fats and aim for total fat intake as follows [4]. For those with TG 200–499 ​mg/dL, aim for 30–35% of energy. If TGs are 500–999 ​mg/dL, total fat intake can be reduced to 20–35% of energy. If TGs are >1000 and in selected patients with TG 500–999 ​mg/dL, a very-low-fat diet (<15% of energy) is recommended to prevent chylomicronemia due to lipoprotein lipase (LPL) deficiency [4]. In patients with high TG and DM, dietary carbohydrate should be lower to prevent hyperglycemia when reducing fat intake. Medium chain TG oil may be used as a source of energy as it will not induce chylomicron production. For patients without LPL deficiency, dietary fat may be liberalized (20–35% of energy) with monitoring of TG response once TG levels reach <500 ​mg/dL [4].
• Include fiber-rich whole grains, vegetables, fruits, low-fat or non-fat dairy products, fatty fish, poultry, tofu, soybeans, lentils and legumes [[2], [3], [4]].
• Abstain or limit alcohol intake, as alcohol can raise TG levels [[2], [3], [4]]. High alcohol intake is associated with elevated TG, especially when obesity is present. Patients with hypertriglyceridemia should be advised to reduce or eliminate alcohol. Complete abstinence is recommended in patients with TG ​≥ ​500 ​mg/dL [[2], [3], [4]].
• Aim for regular physical activity such as walking for a minimum of 30 ​min on most days of the week [[2], [3], [4]].
• Addition of an omega-3 supplement that provides 2 to 4 ​gm/d (EPA ​+ ​DHA) may confer an additional TG reduction [[2], [3], [4],^56^,^57^].

**Table 2:** AHA Classification of triglycerides [57].

• Normal TG ​< ​150 ​mg/dL
• Borderline TG ​= ​150–199
• High TG (HTG) ​= ​200–499
• Very high TG (VHTG) ​= ​500+

The REDUCE-IT (Reduction of Cardiovascular Events with Icosapent Ethyl-Intervention Trial) multicenter randomized double blind placebo controlled trial in 8179 subjects (70.7% for secondary prevention of cardiovascular events) followed for 4.9 years, prescription dose of 4 ​g/day of n-3 fatty acids (FA) (icosapent ethyl) reported a TG reduction of ≥30% in statin treated subjects with TG ​≥ ​500 ​mg/dL and a 20–30% reduction in subjects with TG 200–499 ​mg/dL [57]. The authors concluded that among patients with elevated TG levels despite the use of statins, the risk of ischemic events, including cardiovascular death, was significantly lower among those who received 2 ​g of icosapent ethyl twice daily than among those who received placebo [57]. Evidence whether marine omega 3 fatty acids supplementation lowers risk for MI (myocardial infarction), CHD death, total CHD, CVD death, and total CVD is discussed later.

## 8 Strategy 7: Understand the impact of saturated fats, trans fats, omega-3 and omega-6 polyunsaturated fats and monounsaturated fats on ASCVD risk

Saturated fatty acids (SFAs) and trans fatty acids exhibit the greatest adverse effect on atherogenic cholesterol levels and should be replaced with unsaturated fats, both PUFA and MUFA, from plant sources [[1], [2], [3], [4],^58^]. The ACC/AHA recommend that foods high in saturated fats (e.g., meat, full-fat dairy products, and tropical oils [coconut and palm oil]) should be limited to achieve <7% of energy from SFA [[1], [2], [3], [4]]. In a 2000-kcal dietary pattern, this would translate to 120 ​kcal per day and 13 ​gm of saturated fat. In a 1600-kcal diet pattern, this would translate to 11 ​gm per day [4,30]. The ACC/AHA and the NLA guidelines recommend intake of trans fats should be avoided to reduce ASCVD risk [[2], [3], [4]].

Wang et al. [59] investigated a large cohort with 83,349 women from the Nurses’ Health Study (July 1, 1980, to June 30, 2012) and 42,884 men from the Health Professionals Follow-up Study (February 1, 1986, to January 31, 2012) who were free of CVD, cancer, and DM at baseline. Dietary fat intake was assessed at baseline and updated every 2–4 years [58]. Replacing 5% of energy from saturated fats with equivalent energy from PUFA and MUFA was associated with estimated reductions in total mortality of 27% (HR, 0.73; 95% CI, 0.70–0.77) and 13% (HR, 0.87; 95% CI, 0.82–0.93), respectively [59].

In summary, current evidence [60] supports that different types of dietary fatty acids have divergent effects on CVD risk, and the effects also depend strongly on the replacement macronutrient. A significant reduction in CVD risk can be achieved if SFAs are replaced by unsaturated fats, especially PUFA. Intake of trans fat is consistently associated with higher CVD risk. Both n-6 and n-3 PUFA are associated with lower CVD risk [60]. The 2020–25 DGAC report [1], the ACC/AHA [2,3], the NLA [4] and the 2015–2020 DGA [17] recommend a greater emphasis on types of dietary fats than total amount of dietary fat and recommend replacing foods high in saturated fats with foods high in unsaturated fats, especially PUFA for CVD prevention [[1], [2], [28], [3], [4],^28^].

### 8.1 Saturated fat controversy

Recently there has been a controversy about SFA intake and CVD risk [61,62]. It is well established that saturated fat intake can raise LDL-C levels by downregulating LDL receptors [[1], [2], [3], [4]]. The degree to which LDL-C is impacted by saturated fat intake may also be influenced by other factors, including dietary cholesterol intake, differences in individual SFAs, and individual variances in response to diet [[1], [2], [3], [4]]. In some meta-analysis examining the association of SFA intake and CVD risk, the replacement nutrient was not reported [[63], [64], [65]]. It is likely that study participants’ intake of refined carbohydrates (refined grains and added sugars) may have increased [[1], [2], [3], [4]]. When dietary saturated fat is replaced with refined carbohydrates, LDL-C levels may decrease, but triglycerides and atherogenic small, dense LDL particles may increase while HDL-C decreases, thus not improving overall CVD risk [[1], [2], [3], [4]]. In the Astrup et al. meta-analysis [63] one reason for failure to show an effect of reducing SFA on mortality (RR 0.96; 95% CI 0.90 to 1.03; 11 trials, 55,858 participants) or CV mortality (RR 0.95; 95% CI 0.80 to 1.12, 10 trials, 53,421 participants) could have been the authors did not consider the relatively short period of time for observing a mortality benefit in the included studies.

Hooper et al. conducted a Cochrane systematic review and meta-analysis [66] of RCTs that reported reducing SFA significantly decreased the relative risk of combined CV events by 21%, (risk ratio (RR) 0.79; 95% confidence interval (CI) 0.66 to 0.93,11 trials, 53,300 subjects, 8% with a CV event, I^2^ ​= ​65%, GRADE moderate-quality evidence). Additionally, a meta-regression analysis [66] demonstrated that with greater reductions in SFA consumption (reflected in larger reductions in serum cholesterol), there were greater reductions in the risk of combined CV events. The authors also omitted two large U.S. prospective cohort studies (1980s–2012) with repeated follow-up, which reported that replacing SFA with unsaturated fat was associated with significant reductions in CV mortality and total mortality [59]. Furthermore, the authors did not report that the Hooper et al. meta-analysis included the large Women’s Health Initiative (WHI) interventional study that randomized subjects to a diet low in total fat and not just saturated fat, thus underestimating the overall effect of saturated fat reduction on CVD incidence and mortality [66]. Finally, in the Hooper et al. analyses, there was little or no effect of reducing SFA on non-fatal myocardial infarction or CHD mortality, which could be explained by the small number of these events reported, suggesting the analysis was underpowered to detect the benefit [63,66]. The authors did not include an important meta-analysis conducted as part of the AHA Presidential Advisory on Dietary Fats and Cardiovascular Disease [58] that supported replacing SFA with PUFA.

The PURE (Prospective Urban Rural Epidemiological) study [67] reported that in 135,000 people (mostly without CVD) from 18 countries in five continents (80% low- and middle-income countries), an increase in consumption of all types of fat (SFA, MUFA and PUFA) was associated with lower risk of death and had a neutral association with CVD. A diet high in carbohydrate was associated with higher risk of death, but not with risk of CVD. Several flaws in the PURE study prompted editorials that cautioned against applying its findings, derived primarily from low-income countries, to make inferences about dietary intakes in high-income countries [68]. The macronutrient distributions in the PURE study deviated significantly from those in the United States. The highest quintile in this cohort consumed 77% of kcal from carbohydrate, while the lowest quintile consumed 46% (reflective of average American carbohydrate intake). Similarly, SFA intake was 2.8% of kcal in the lowest quintile and 13% in the highest quintile. SFA intake consistent with the lowest quintile would rarely be observed in a healthy person and is likely reflective of severe malnutrition or critical illness, thus limiting extrapolation to the U.S. or other high-income countries. Furthermore, other factors such as differences in poverty levels, access to medical care, and food insecurity could have confounded these results [68]. Points of concurrence and disagreement regarding the saturated fat controversy and list of identified research needs are listed in Table 3 [61,62].

**Table 3:** Types and sources of fats and their effect on serum lipids [[1], [2], [3], [4]].

1. Monounsaturated fat (omega-9) may lower LDL-C and ASCVD risk. • Extra virgin olive oil, canola oil, peanut oil • Avocados, olives (very high in sodium) • Unsalted nuts: almonds, peanuts, pecans, pistachios, hazelnuts
2. Polyunsaturated fat (omega- 6 and plant omega 3): help lower LDL-C when they replace saturated fat. • Omega 6 Linoleic acid: Corn oil, safflower oil, sunflower oil, soybean oil, sunflower seeds. • Omega 3 Alpha-linolenic acid: Flax seed oil, canola oil, soy bean oil, English walnuts, edamame, hemp seeds, chia seeds, flax seeds and fenugreek seeds.
3. Saturated fats raise LDL-C. Saturated fats should be avoided or eaten in small amounts. Saturated fats are solid at room temperature. • Fatty cuts of lamb, pork, beef, poultry with skin, beef fat, lard, bacon, sausage, hotdogs. • Whole milk & whole milk products: butter, ghee, cheese, cream, ice-cream, yogurt made from whole milk. • Palm oil, palm kernel oil and coconut oil and coconut cream
4. Trans fats: raise LDL-C and CVD risk and should be avoided if they are labeled as partially hydrogenated fats. • Baked goods: pastries, cakes, donuts, cookies. • Fried foods: French fries, fried chicken, onion rings and deep-fried snacks cooked in re-used oil. • Stick margarine, shortening • Butter, meat, cheese and dairy products

### 8.2 Red meat and processed meat

While red meat, including beef, pork, and lamb, contributes beneficial nutrients such as protein, iron, and zinc, high intakes of both unprocessed and processed red meats have been linked to increased risk of CVD. Several factors are thought to increase CVD risk. Red meat is high in saturated fat and cholesterol, and also in l-carnitine and phosphatidylcholine (which is broken down to choline, found abundantly in eggs), both used in the formation of the pro-atherogenic metabolite trimethylamine-N-oxide (TMAO) by gut microbes [69].

High plasma levels of TMAO are linked to increased risk of CVD and incident major adverse cardiac events [70]. Replacing 2 servings a day of red meat with plant-based protein sources for 8 weeks significantly reduced TMAO and LDL-C levels [40]. Further, red meat is the primary source of heme iron, with high iron stores thought to contribute to oxidative stress through the promotion of LDL oxidation [69,71]. A significant positive association was reported between red meat intake, iron load (measured by plasma ferritin), and myocardial infarction (MI) risk [72].

Processed red meats are more strongly linked to CVD risk than are unprocessed red meats [[1], [2], [3], [4],^73^]. A meta-analysis examining unprocessed and processed red meat intake and incident CHD (including 20 studies totaling 1,218,380 individuals) found that processed red meats are associated with a 42% increased risk of CHD per each 50 ​gm serving per day [73]. This analysis did not find an association between unprocessed red meat intake and CHD [73].

However, the Nurses’ Health Study followed 84,136 women over 26 years, and did observe an increased risk of CHD in those with higher intake of unprocessed red meat. Furthermore, when compared to red meat consumption, intake of nuts, fish, poultry, and low-fat dairy were associated with a significantly reduced risk of CHD [74]. A meta-analysis of 13 cohort studies (1,674,272 individuals) examined the association between meat consumption and CVD mortality, finding that those with the highest intake of processed meat and unprocessed red meat had 18% and 16% higher risk of CVD mortality, respectively [75]. A recent study in 43 adults reported that substituting 150 ​gm/day of lean, unprocessed beef for carbohydrate in a Healthy U.S.-Style Eating Pattern resulted in a shift toward larger, more buoyant LDL subfractions, but otherwise had no significant effects on the cardiometabolic risk factor profile in adult men and women with prediabetes and/or metabolic syndrome [76]. In totality, the evidence indicates that, when considering CV health, processed red meats should be avoided, while unprocessed meats should be limited and replaced with more protein foods that benefit CVD health such as nuts, legumes, fish, and poultry [[1], [2], [3], [4]]. Red and processed meat are also associated with increased inflammatory biomarkers [77].

### 8.3 Omega-3 fatty acids, fish intake and omega-6 fatty acids

A recent meta-analysis of 13 trials reported that during a mean treatment duration of 5.0 years, 3838 myocardial infarctions, 3008 CHD deaths, 8435 total CHD events, 2683 strokes, 5017 CVD deaths, 15,759 total CVD events, and 16,478 major vascular events were documented. In the analysis excluding REDUCE-IT (Reduction of Cardiovascular Events with Icosapent-Ethyl Intervention Trial), marine omega-3 supplementation was associated with significantly lower risk of myocardial infarction (rate ratio [RR] [95% CI]: 0.92 [0.86, 0.99]; P ​= ​0.020), CHD death (RR [95% CI]: 0.92 [0.86, 0.98]; P ​= ​0.014), total CHD (RR [95% CI]: 0.95 [0.91, 0.99]; P ​= ​0.008), CVD death (RR [95% CI]: 0.93 [0.88, 0.99]; P ​= ​0.013), and total CVD (RR [95% CI]: 0.97 [0.94, 0.99]; P ​= ​0.015). Statistically significant linear dose–response relationships were found for total CVD and major vascular events in the analyses with and without including REDUCE-IT. Despite excluding the REDUCE-IT trial which used mineral oil as a placebo that interferes with statin absorption, the authors concluded that marine omega-3 supplementation lowers risk for myocardial infarction, CHD death, total CHD, CVD death, and total CVD. Further, risk reductions appeared to be linearly related to marine omega-3 dose [78].

Of note, following the recommendation from an independent Data Monitoring Committee the STatin Residual Risk Reduction with EpaNova in HiGh CV Risk PatienTs with Hypertriglyceridemia (STRENGTH) trial, a large-scale, global CV outcomes trial was discontinued due to its low likelihood of demonstrating a benefit to patients with mixed dyslipidemia who are at increased risk of CVD. It was designed to evaluate the safety and efficacy of *Epanova (o*mega-3 carboxylic acids) compared to placebo, both in combination with standard-of-care statin medicines (https://www.astrazeneca.com/media-centre/press-releases/2020/update-on-phase-iii-strength-trial-for-epanova-in-mixed-dyslipidaemia-13012020.html).

Higher intakes of n-3 and n-6 fatty acids were associated with the lowest levels of inflammatory biomarkers [79]. Increased intake of n-6 fatty acids did not lead to increased pro-inflammatory cytokines e.g. CRP, IL-6, and soluble TNF receptors 1 and 2 [79]. Both n-3 and n-6 fatty acids were inversely associated with pro-inflammatory interleukin-1Ra and positively associated with anti-inflammatory transforming growth factor-β [79]**.**

Types and sources of fats and their effects on serum lipids are listed in Table 4.

**Table 4:** Points of concurrence and disagreement regarding saturated fat controversy and list of identified research needs [62].

Points of concurrence	Points of disagreement	Research needs Identified
• Currently recommended healthy food-based eating patterns are not high in SFAs (<10% of energy). • Mediterranean diet intervention trials demonstrate the importance of a dietary pattern where overall dietary composition, beyond just individual nutrients (such as SFAs), can lower CVD risk. • Advice to maximally reduce SFAs can have unintended consequences if implementation is done inappropriately with respect to the nutrients and foods that are substituted. • For reducing elevated LDL-cholesterol concentrations, it is widely recommended that dietary SFAs be decreased. • LDL cholesterol lowering in response to decreasing SFA intake can vary significantly among individuals. • Individual SFAs have differing biological effects. • The food matrix can affect the LDL-cholesterol response to SFAs.	• Does lowering SFA intake reduce the incidence of CVD? • To what extent is the LDL-cholesterol reduction with lower SFA intake predictive of reduced CVD risk? • Do dietary SFAs importantly affect factors other than LDL cholesterol that may impact CVD risk? • Is there clear rationale for setting a target for maximally reducing dietary SFAs? A second question centers around interpreting the impact on CVD of LDL-cholesterol reductions achieved by reducing SFA intake. While there is agreement that LDL-cholesterol concentrations are related to CVD risk to a significant degree, there remains disagreement as to the extent that the reduced LDL cholesterol that is achieved with lower SFA intake reflects the impact of atherogenic particles, including remnant lipoproteins and small dense LDL. There is also concern that concomitant decreases in HDL cholesterol could represent a reduction of the cardioprotective effects of HDL particles. Third, there is a lack of agreement as to the rationale for setting a maximally reduced dietary SFA target for population guidelines. It is argued that such a target allows for inclusion of select individual foods that are high in SFAs within an overall healthy diet. On the other hand, there is concern as to the scientific basis for implementing a numerical population-wide target for dietary SFAs (e.g., <10%, <7%, or lower). Such targets may fail to consider evidence that different SFAs and food sources of SFAs, as well as varying individual responses, can have differing effects on cardiometabolic risk. Finally, we would suggest that a number of the controversies outlined in this debate, as well as other important questions regarding the health effects of SFAs, including those that extend beyond CVD risk, could be resolved by achieving a much stronger evidence base than currently exists. Some key evidence gaps are enumerated in Column 3. We hope that findings derived from novel research addressing these needs will lead to more effective dietary guidelines and enhance the implementation of dietary practices aimed at improving and maintaining the health of the population.	• Determine effects on cardiometabolic risk factors of interactions of specific SFAs with other dietary factors, particularly the amount and type of carbohydrate, in healthy individuals as well as those at high risk (e.g., with increased adiposity or glucose intolerance). • Evaluate potential racial and ethnic differences in response of cardiometabolic risk factors to variation in SFA intake. • Examine the long-term relations between healthful dietary patterns differing in SFA content worldwide and morbidity/mortality outcomes, taking into account LDL cholesterol and other risk factors. • Identify laboratory measures or imaging studies that can provide more reliable surrogates for CVD outcomes than those currently in use, and hence may minimize the need for long-term CVD outcome studies. • Determine dose–response of SFAs on cardiometabolic risk factors, both under isocaloric conditions (with substitution of other macronutrients) and with overfeeding. • Identify genomic and epigenomic factors, as well as variations in the microbiome, that may contribute to interindividual variation in effects of SFAs on cardiometabolic risk factors. • Investigate more extensively the effects of individual SFAs and SFA-rich foods (and the nutrients/foods that are substituted for them) on insulin/glucose, inflammation, thrombosis, and brain health, as well as other chronic diseases. • Evaluate effective implementation strategies for achieving adherence to food-based dietary recommendations.

### 8.4 Fish intake

Compared to little or no intake, two servings (6–8 oz) per week of fatty fish or seafood providing 250–500 ​mg/day of marine n-3 PUFA, i.e., eicosapentenoic acid (EPA) and dexahexanoic acid (DHA), was associated with a 36% decreased risk of CHD mortality. A 10% reduction in CVD risk was reported in plant n-3 alpha linolenic acid (ALA) intake studies between the highest vs. lowest tertile of intake. Only 0.2–8% of ALA is converted to EPA [59]. An ALA intake of 0.6–1.2% of energy is recommended ( [[2], [3], [4],^28^].

## 9 Strategy 8: Limit excessive intake of dietary cholesterol for those with dyslipidemia, diabetes and at risk for heart failure

The NLA [4] recommends a diet containing reduced amounts of cholesterol (<200 ​mg/day) to decrease LDL-C and non–HDL-C, especially for persons at high risk for CVD, including those with diabetes [4]. The 2019 ACC/AHA Guideline [2] on the Primary Prevention of Cardiovascular Disease cited studies of the benefit of plant-based rather than animal-based protein and concluded, “A diet containing reduced amounts of cholesterol can be beneficial to decrease atherosclerotic CVD risk” [2]. The 2019 AHA Advisory [29] recommends that “patients with dyslipidemia, particularly those with diabetes mellitus or at risk for heart failure, should be cautious in consuming foods rich in cholesterol.” Dietary cholesterol modestly increases total-C and LDL-C on average, with each 100 ​mg dietary cholesterol consumed per day increasing LDL-C by 1.93 ​mg/dL, although hyper- and hypo-responders exist. Heart-healthy dietary patterns with a relatively high ratio of polyunsaturated fats to saturated fats are typically low in dietary cholesterol, while choosing plant-based protein sources also limits cholesterol intake [4,18,29].

Due to the relatively high content of cholesterol in egg yolks, it remains advisable to limit intake. A large 2019 study following 29,615 adults over 17.5 years found a significant association between higher intake of eggs or dietary cholesterol – in a dose-dependent manner – and the risk of incident CVD and all-cause mortality [80]. Interestingly, these findings were independent from overall diet quality, suggesting that even those adhering to healthier eating patterns will benefit from reducing intake of dietary cholesterol and egg yolks [80]. A 3-oz serving of shrimp has an equivalent amount of cholesterol as a whole egg, however, shrimp and other shellfish are otherwise quite low in saturated fat and have minimal effects on raising blood cholesterol. Thus, shellfish or eggs can be part of a heart-healthy dietary pattern when paired with other lean animal-based or plant-based protein sources [81].

In summary, the AHA recommends that healthy individuals may include up to a whole egg or equivalent daily with the following exceptions [29,81]:

• **Vegetarians (lacto-ovo)** who do not consume meat-based cholesterol-containing foods may include more dairy and eggs in their diets in moderation [1,29,81]**.**
• **Patients with dyslipidemia**, particularly those with diabetes mellitus or at risk for heart failure, should be cautious in regularly consuming foods rich in cholesterol [4,29]**.**
• **For older normocholesterolemic patients**, given the nutritional benefits and convenience of eggs, consumption of up to 2 eggs per day is acceptable within the context of a heart-healthy dietary pattern [1,29,81].

## 10 Strategy 9: Include dietary adjuncts such as viscous fiber, plant sterols/stanols and probiotics

A growing body of evidence indicates that functional foods including herbs, spices and dietary supplements have physiological benefits and reduce the risk of ASCVD [31,82,83]**.**

• **Viscous fiber:** Viscous fiber such as beta glucans, pectin, gums and mucilage reduce LDL-C. The NLA recommends 5–10 ​g of viscous fibers per day or even greater as tolerated [4]. This amount will reduce LDL-C by 4–10% [4]. Food sources of viscous fiber include oats, barley, legumes, lentils, apples, pears, plums, oranges, broccoli, Brussels sprouts, carrots and peas. Supplements are available as fiber laxatives and contain psyllium and methyl cellulose [4].
• **Plant sterols/stanols:** The NLA recommend 2 ​g/day dose of plant sterols/stanols, also referred to as phytosterols (PS), and will reduce LDL-C by 5–10% [4]. A typical Western diet provides 200–400 ​mg/day and a vegan diet provides 400–800 ​mg/day. PS lower LDL-C by competing with dietary cholesterol for absorption. The predominant plant sterols and stanols in our habitual diets are sitosterol (66%), campesterol (22%), stigmasterol (8%), and sitostanol plus campestanol (4%) which are present in bread, cereals, vegetables, fruits and (vegetable) oils and products based on these oils [84]. Plant sterols and stanols are present mainly as fatty acid esters, hydroxycinnamic acid esters, and glycosides [84]. An increase in the intracellular PS level upregulates the adenosine triphosphate-binding cassette transporter (ABC) G5/G8 to move all sterols including cholesterol out of the enterocytes and into the lumen. Within the gastro-intestinal tract, all ester bonds are cleaved by specific enzymes, resulting in the formation of free plant sterols and stanols. The free plant sterols and stanols are subsequently incorporated into mixed micelles, a process in which they interfere with cholesterol incorporation into these micelles [84]. PS are available in fortified foods/beverages and supplements [4].

The lipid-lowering impact of PS-fortified products (both free plant sterols and stanols and their esterified forms) for lowering atherogenic cholesterol was similar in response to fat versus non-fat foods, dairy vs. non-dairy foods at intakes ≤ 2g/day [4,[82], [83], [84]]. PS have extremely low bioavailability (0.5%–2%) and are rapidly excreted by the liver vs 55%–60% of exogenous cholesterol [[82], [83], [84]]. To achieve maximal efficacy, foods or supplements containing PS should be taken during main meals, when cholesterol present in the gut lumen is higher than in the fasting state due to the stimulation of biliary secretions containing cholesterol and to the dietary cholesterol derived from food [4,[82], [83], [84]]. An increased intake of carotenoid-rich fresh fruit and vegetables are recommended due to a reduced absorption of fat-soluble vitamins (carotenoids) following intake of plant sterol/stanol-enriched food such as tub spreads [4,[82], [83], [84]].

PS can be safely consumed in combination with statin medications to aid LDL-C lowering [4,[82], [83], [84]]. Persons with sitosterolemia should avoid foods fortified with PS. It is noteworthy that genetic variations in cholesterol/phytosterol absorption can affect levels of circulating non-HDL-C and risk of CAD. A recent study examined the effects of ABCG5/8 variants on non-HDL-C (N 610532) and phytosterol levels (N ​= ​3039) and reported that both dietary cholesterol and phytosterols contribute directly to atherogenesis. The study concluded that in individuals with rare ABCG5/8 coding variants who have increased non-HDL-C levels and elevated phytosterol levels, there is a 2-fold increased risk of CAD [85].

• **Nutraceuticals and dietary supplements:** Nutraceuticals may be an additional option for patients who cannot tolerate statins because of severe muscle pain. Emerging evidence shows bergamot, berberine, artichoke leaf extract, red yeast rice (RYR), soluble fiber, and plant stanols/sterols as monotherapy or adjunctive therapy may be effective at lowering LDL-C [82,83].

In an RCT [86] conducted in China, RYR extracts (xuezhikang) with an average content of 2.5–3.2 ​mg of monacolin, administered to a population of about 5000 elderly subjects with previous coronary events such as a myocardial infarction (China Coronary Secondary Prevention Study), led to a 20% reduction in LDL cholesterol levels, compared to placebo [86]. The cholesterol-lowering effect was associated with a significant decrease of fatal and non-fatal coronary events, stroke and all-cause mortality (−31%, −44% and −32% respectively) over the four years duration of the trial [86]. Combining statins with RYR-based supplements is discouraged for pharmacodynamic reasons (both have the same mechanism of action) and comparable side effects [82,83].

In many of the RYR supplements, the amount of monacolin is now 10 ​mg, likely due to the European Food Safety Authority’s (EFSA) approval of the claim of “maintenance of normal cholesterol values” at this dose exclusively (82). Considering the widespread availability of RYR supplements, the absolute incidence of the related adverse side effects is rather low [82]. However, medical supervision is important for the use of supplements, especially with regards to possible interactions between RYR and other drugs, and for the selection of appropriate patients for this treatment [82]. Spices such as turmeric (curcumin), cinnamon and fenugreek seeds may also exhibit anti-inflammatory effects [31]**.**

• **Probiotics:** Non-fat or low-fat yogurt without added sugar can be an important nutritional component of a heart-healthy diet. A review of 26 clinical studies and two meta-analysis of multiple probiotic strains reported both L. reuteri significantly lowered LDL-C by 8.9–11.6% and E. faecium lowered LDL-C by 5% [31,83,87]. L. reuteri was provided in yogurt or capsule form and significantly lowered LDL-C and inflammatory markers versus placebo [31,83,87]. More research is needed to recommend probiotics as a supplement to improve the lipid profile [31,83].
• **Caffeine:** Caffeine intake has historically been linked to higher rates of hypertension (HTN) and CVD, though current evidence shows that, for habitual caffeine consumers, neither appear to be increased over the long-term [88,89]. For novel caffeine consumers, intake does cause a short-term BP increase similar to the impact on normotensive subjects [89]. Long-term intake of coffee, over 2 weeks or more, does not appear to have a substantial impact on BP, neither in normotensive nor in hypertensive individuals [89,90].

Coffee is by far the primary source of caffeine in the U.S [88]. Unfiltered coffee – such as French press, Scandinavian boiled coffee, or Turkish coffee – contains a diterpene called cafestol that produces a lipid-raising effect [91]. High intake of unfiltered coffee (6 cups per day) has been shown to increase LDL-C by 17.8 ​mg/dL. Cafestol is removed when brewing coffee through a paper filter, thus, filtered coffee does not have a significant impact on LDL-C levels [89,92].

A recent study [93] reported that moderate consumption of caffeinated energy drinks corresponding to an acute caffeine intake of up to 200 ​mg did not result in clinically relevant cardiovascular changes in young healthy adults. However, high intake of an energy drink (about 1 ​L) was associated with moderate to severe adverse effects in some participants (e.g., prolonged QT interval, palpitations) [93]. Overall, moderate caffeine intake, including filtered coffee, does not appear to increase risk of CVD, and may actually provide protective benefits. In a dose-response meta-analysis of the relationship of coffee intake and CVD risk (36 studies with 1,279,804 participants), moderate coffee consumption, defined as 3 to 5 cups per day, was associated with the lowest CVD risk, while heavy consumption did not increase CVD risk compared to non-consumers [94].

## 11 Strategy 10: Implement AHA/ACC [2] and NLA [4] physical activity recommendations for the optimization of lipids and prevention of ASCVD

• Minimum of 150 ​min per week of moderate intensity or 75 ​min per week of higher intensity aerobic activity for lowering TG and ASCVD risk and raising HDL-C e.g. biking, running, speed walking and swimming at 40–75% aerobic capacity. For adults unable to meet the minimum physical activity recommendations, engaging in some moderate- or vigorous-intensity physical activity, even if less than this recommended amount, can be beneficial to reduce ASCVD risk [4].
• >2000 ​kcal/week of physical activity (200–300 ​min per week) for lowering LDL-C, body weight and body fat [4].
• Resistance exercise to maintain strength, balance, and bone density, e.g., weight training or resistance training. Reducing sedentary behavior in adults may be reasonable to reduce ASCVD risk [4].

The ACC/AHA Exercise and Physical Activity Recommendations [2] are listed in Table 5. The ACC/AHA definitions and examples of different intensities of Physical Activity are noted in Table 6.

**Table 5:** ACC/AHA exercise and physical activity recommendations [2].

Recommendations for Exercise and Physical Activity		
COR	LOE	Recommendations
I	B-R	1. Adults should be routinely counseled in healthcare visits to optimize a physically active lifestyle.
I	B-NR	2. Adults should engage in at least 150 ​min per week of accumulated moderate-intensity or 75 ​min per week of vigorous-intensity aerobic physical activity (or an equivalent combination of moderate and vigorous activity) to reduce ASCVD risk.
IIa	B-NR	3. For adults unable to meet the minimum physical activity recommendations (at least 150 ​min per week of accumulated moderate-intensity or 75 ​min per week of vigorous-intensity aerobic physical activity), engaging in some moderate- or vigorous-intensity physical activity, even if less than this recommended amount, can be beneficial to reduce ASCVD risk.
IIb	C-LD	4. Decreasing sedentary behavior in adults may be reasonable to reduce ASCVD risk.

**Table 6:** Definitions and examples of different intensities of physical activity [2].

Intensity	METs	Examples
Sedentary behavior∗	1–1.5	Sitting, reclining, or lying; watching television
Light	1.6–2.9	Walking slowly, cooking, light housework
Moderate	3.0–5.9	Brisk walking (2.4–4 mph), biking (5–9 mph), ballroom dancing, active yoga, recreational swimming
Vigorous	≥6	Jogging/running, biking (≥10 mph), singles tennis, swimming laps

## 12 Conclusion

Research has shown that medical nutrition therapy by an RDN over multiple visits led to improved lipids, weight, HbA1c and high blood pressure along with cost savings. A collaborative team-based approach includes referral to an RDN to help patients achieve their nutrition goals and to provide support and accountability. Adults should eat a heart-healthy diet which emphasizes plant-based foods such as vegetables, fruits, legumes, nuts, whole grains, and also lean protein foods and fish. Limit foods high in saturated fats and dietary cholesterol and reduce sodium (salt). Avoid *trans*-fat, processed meats, refined carbohydrates and sugar-sweetened foods and beverages. Include dietary adjuncts such as viscous fiber, plant sterols/stanols, and long-chain omega-3 fatty acids. Although some nutraceuticals have been shown to significantly improve the efficacy of standard pharmacological treatments, more research is needed as no outcome studies are available proving that nutraceuticals can prevent CVD morbidity or mortality. Weight loss of 5%–10% of initial weight, if needed, achieved through comprehensive lifestyle intervention, has been shown to improve BP, delay the onset of T2DM, improve glycemic control in T2DM, and improve the lipid profile. Nutrition resources for health care practitioners and patients from health care organizations are listed in Table 7.

**Table 7:** Nutrition Resources for health care practitioners and patients from health care organizations.

American College of Cardiology
https://www.cardiosmart.org/nutrition/
https://www.cardiosmart.org/News-and-Events/2018/08/Strategies-for-Improving-Diet-and-Improving-Heart-Health
https://www.cardiosmart.org/∼/media/Documents/Infographics/Heart-Healthy%20Diets.ashx/
American Heart Association
https://www.heart.org/en/healthy-living/healthy-eating/eat-smart/nutrition-basics
https://www.cardiosmart.org/nutrition
Academy of Nutrition and Dietetics
Find a nutrition experthttps://www.eatright.org/find-an-expert
https://www.eatright.org/-/media/eatright-files/nationalnutritionmonth/handoutsandtipsheets/nutritiontipsheets/2020/20healthtipsfor2020_nnm20_final.pdf
National Lipid Association Clinical Lifestyle Modification Tool (CLMT) Kit www.lipid.org/CLMT
• Heart-Healthy Eating: Southern Style
• Heart-Healthy Eating: DASH Style
• Building a Heart Healthy Plate
• Heart-Healthy Eating On A Budget
• Heart-Healthy Eating if You Are Underweight
• Heart-Healthy Eating: Mediterranean Style
• Heart Healthy Eating: Latino Style
• Heart-Healthy Eating: Asian/Indian Style
• Heart-Healthy Eating: Vegetarian Style
• Let’s Eat for the Health of it. Choose MyPlate.gov. www.cnpp.usda.gov/Publications/Myplate/DG2010Brochure.pdf
• Heart and Vascular Diseases, Detailed Information on Cholesterol, Heart Attack, High Blood pressure, Obesity, Other Heart and Vascular Diseases. www.nhlbi.nih.gov.health/prof/heart/index.htm
• What is a standard drink? https://www.niaaa.nih.gov/what-standard-drink.
• Calorie Results and Food Tracking Worksheets. www.choosemyplate.gov/professionals/food_tracking_wksht.html
• Your Guide to Lowering Blood Pressure with DASH. www.nhlbi.nih.gov/helath/public/heart/hbp/dash/new_dash.pdf
• FDA Consumer Updates http://www.fda.gov/ForConsumers/ConsumerUpdates/ucm372915.htm

## Declaration of competing interest

The authors declare no conflict of interest.
